# Quantitative Evaluation of Stomatal Cytoskeletal Patterns during the Activation of Immune Signaling in *Arabidopsis thaliana*

**DOI:** 10.1371/journal.pone.0159291

**Published:** 2016-07-14

**Authors:** Masaki Shimono, Takumi Higaki, Hanae Kaku, Naoto Shibuya, Seiichiro Hasezawa, Brad Day

**Affiliations:** 1 Department of Plant, Soil, and Microbial Sciences, 1066 Bogue Street A286, Michigan State University, East Lansing, Michigan, 48824, United States of America; 2 Department of Integrated Biosciences, The University of Tokyo, Kashiwanoha 5-1-5, Kashiwa, Chiba, 277–8562, Japan; 3 Department of Life Sciences, School of Agriculture, Meiji University, 1-1-1 Higashimita, Tama-Ku, Kawasaki, 214–8571, Japan; 4 Graduate Program in Genetics, 2240E Biomedical Physical Sciences, Michigan State University, East Lansing, Michigan, 48824, United States of America; 5 Graduate Program in Cell and Molecular Biology, 2240A Biomedical Physical Sciences, Michigan State University, East Lansing, Michigan, 48824, United States of America; UMBC, UNITED STATES

## Abstract

Historically viewed as primarily functioning in the regulation of gas and water vapor exchange, it is now evident that stomata serve an important role in plant immunity. Indeed, in addition to classically defined functions related to cell architecture and movement, the actin cytoskeleton has emerged as a central component of the plant immune system, underpinning not only processes related to cell shape and movement, but also receptor activation and signaling. Using high resolution quantitative imaging techniques, the temporal and spatial changes in the actin microfilament array during diurnal cycling of stomatal guard cells has revealed a highly orchestrated transition from random arrays to ordered bundled filaments. While recent studies have demonstrated that plant stomata close in response to pathogen infection, an evaluation of stimulus-induced changes in actin cytoskeletal dynamics during immune activation in the guard cell, as well as the relationship of these changes to the function of the actin cytoskeleton and stomatal aperture, remains undefined. In the current study, we employed quantitative cell imaging and hierarchical clustering analyses to define the response of the guard cell actin cytoskeleton to pathogen infection and the elicitation of immune signaling. Using this approach, we demonstrate that stomatal-localized actin filaments respond rapidly, and specifically, to both bacterial phytopathogens and purified pathogen elicitors. Notably, we demonstrate that higher order temporal and spatial changes in the filament array show distinct patterns of organization during immune activation, and that changes in the naïve diurnal oscillations of guard cell actin filaments are perturbed by pathogens, and that these changes parallel pathogen-induced stomatal gating. The data presented herein demonstrate the application of a highly tractable and quantifiable method to assign transitions in actin filament organization to the activation of immune signaling in plants.

## Introduction

Actin is perhaps the most functionally versatile protein found in eukaryotes, having both direct and indirect associations with most cellular processes, including those required for development, cell shape and movement, and response to stress [1]. In this regard, it is not surprising that actin’s activity and ubiquity qualify it as the ideal surveillance platform [2], underpinning a vast array of cellular functions, ranging from stimulus perception to signal transduction. To fulfill this role, changes in actin cytoskeletal organization are tightly regulated, a feature that imparts both rapidity and specificity. For example, in mammalian cells, mechanical and immune-associated stressors have been demonstrated to induce the rapid stimulus specific changes in cytoskeletal organization [3]. Similarly, in plants, stimulus-induced changes in the actin cytoskeletal array have also observed, with response to pathogen infection being among the best-studied signaling cascades associated with these changes [1]. Collectively, work in this area has demonstrated that the plant actin cytoskeleton responds to, and is likely required for, host signaling resulting from a variety of biotic stimuli, including those associated with bacterial [4], fungal [5], and viral [6] infection.

The role of the actin cytoskeleton during plant immune activation and signaling has been best defined through studies charactering elicitor-induced changes in cytoskeletal organization following the activation of pattern-triggered immunity (PTI; [7]), a robust immune signaling cascade activated via the recognition of conserved pathogen-associated molecular patterns (PAMPs; e.g., bacterial flagellin, fungal chitin) by plasma membrane-localized pattern recognition receptors (PRRs) [8]. Through these analyses, it has been demonstrated that actin is associated with the activity of a number of cellular processes required for immune signaling, including the redirection and focal accumulation of immune signaling complexes [9], the endocytosis of PRRs [10], the generation of reactive oxygen [11], callose deposition [12], and the modulation of transcription [13]. More recent work has begun to define the complexity of the interactions amongst, and organization within, actin microfilaments (MFs) required for the activation of signaling in response to a variety of stimuli [14–16]. For example, using quantitative imaging approaches, it has been demonstrated that temporal and spatial changes in the rates of actin MF elongation and severing, including changes in MF bundling, parallel the activation of biotic stress signaling [17,18]. To demonstrate that this response is specific and required for the proper activation of immunity, several recent studies have shown that the inhibition of MF dynamics using latrunculin-B, or genetic mutants of a variety of actin binding proteins, blocks the activation of immune signaling following pathogen infection [19,20]. Related to this, more recent work has also shown that the plant actin cytoskeleton is targeted by phytopathogenic bacteria during infection, thus providing the first evidence that plant pathogenic type-III secreted effectors induce perturbations in MF organization to promote infection and disease [21]. In total, these studies demonstrate that actin is both necessary and required for broad-based immune signaling processes associated with phytopathogen virulence and host immunity.

To facilitate the high-resolution spatial and temporal evaluation of actin dynamics during the naïve oscillation of the circadian clock, Higaki et al. developed quantitative methods to classify MF organization in Arabidopsis guard cells [15]. Using hierarchical clustering analysis based on three metrics to quantitatively evaluate MF orientation, bundling, and density, it was observed that guard cell-localized MFs transiently bundled during the stomatal opening process during diurnal cycle, and assume a radial orientation when stomata are fully open [15]. Using this approach, it was revealed that each of the identified MF configurations represent distinct transitions, each of which are required for proper stomata opening and closing patterns. In the current study, we evaluated the temporal and spatial changes of stomatal guard cell MFs during the activation of immune signaling. As presented herein, we collected and analyzed MF configurations from guard cell pairs during bacterial pathogen infection and pathogen-elicitor treatment. We observed that both bacterial infection and diurnal cycling resulted in MF configurations that consisted primarily of longitudinal arrays in closed guard cells. However, and as a function of the relationship between MF organization and the activation of stomatal immunity, we show that a specific change in actin MF configuration in stomatal guard cells following flg22 and [GlcNAc]_7_ (hereafter referred to as chitin) treatments did not lead to the accumulation of MF longitudinal arrays. We posit that pathogens actively alter actin cytoskeletal configurations to evoke physiological changes in their host–in this case, the opening of stomata and closing of stomata. The data presented herein provide new insight into the regulation of stomatal immunity in plants, further establishing actin as an important component of host immunity through the temporal and spatial organization of the stomatal guard cell during pathogen infection.

## Results

### Evaluation of actin cytoskeletal parameters in stomatal guard cells

**Fig 1:**
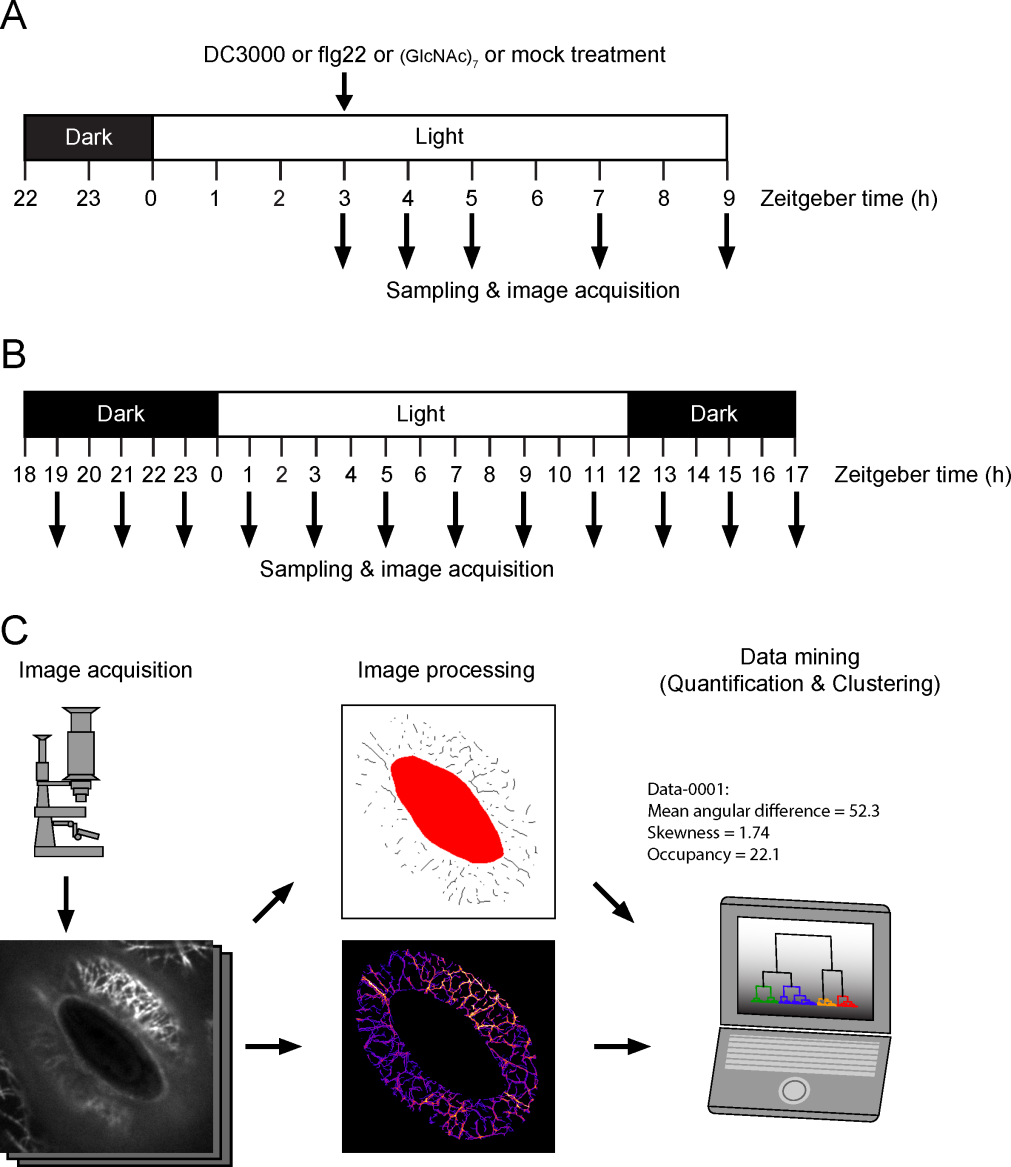
Experimental workflow for the treatment, data collection, and quantitative evaluation of actin microfilament (MF) configurations in guard cells. (A) Illustration of the workflow for image acquisition of Arabidopsis guard cell pairs from mock-, pathogen-, and PAMP-elicited samples. Sampling times are shown as Zeitgeber time, which refers to the experimental time. All experiments were conducted under 12 light/12 hour dark cycles; Zeitgeber time zero indicates the time at which the lights turn on. (B) Illustration of image collection for diurnal image sampling. (C) Guard cell images (n = 1793) collected by spinning disc confocal microscopy were processed and analyzed, and further classified based on MF configurations following immune elicitation (i.e., PAMP treatment, pathogen treatment). Using this approach, we evaluated stomatal aperture and three metric parameters for MF orientation, bundling and density, independently. Based on the three MF configuration parameters, images were classified into some MF configuration classes and time evolution of the class frequency was assessed.

Quantitative imaging of the stomatal guard cell actin cytoskeleton during circadian oscillations has shown that actin filaments undergo regular patterns of organization, a response that tracks the diurnal opening and closing of stomata [15]. To define the temporal and spatial changes in guard cell actin MF organization following phytopathogenic bacteria infection and pathogen elicitor treatment, we imaged 1,793 guard cell pairs from *Arabidopsis thaliana* ‘Columbia’ (Col-0) seedlings expressing the fluorescent actin marker GFP-fABD2 [17]. As illustrated in Fig 1, we used spinning disc confocal microscopy (SDCM) to collect serial sections (0.5 μm intervals) of actin MFs from Arabidopsis guard cells at time points ranging from 0–6 hours post-inoculation (hpi) with the virulent pathogen *Pseudomonas syringae* pv. *tomato* DC3000 (*Pst* DC3000), including from samples treated with the purified pathogen elicitors flg22 and chitin (Fig 1A). In parallel, serial sections of actin MFs in guard cells were collected over a 24 h period (every 2 hours) over the diurnal cycle (12 h light/12 h dark; Fig 1B).

To evaluate induced spatial changes in guard cell actin MF organization during pathogen infection and immune activation, we conducted image quantification and clustering analyses of samples isolated from naïve diurnal cycles and used these as a benchmark for comparison to the induced changes during the activation of plant immunity. As illustrated in Fig 1C, and as previously defined [15], we measured the mean angular differences between MFs and the nearest stomatal pore edge (metrics for MF orientations), the skewness of GFP-fABD2 fluorescence intensity distribution (metrics for MF bundling), and occupancy of skeletonized MF pixels in guard cell regions (metrics for MF density) from the original serial confocal sections collected by SDCM. The mean angular difference has the highest value of 90 degrees when MFs show a perfect radial orientation. Conversely, values lower than 45 degrees represents a longitudinal MF orientation. Skewness of GFP-fABD2 intensity distribution is used for evaluating MF bundling, and its value becomes higher when high intensity pixel numbers are increased by MF bundling. The occupancy becomes higher when MFs become dense. Taken together, the analysis of each of these metrics allows us to evaluate, assign, and correlate the precise spatial orientation of actin MFs, and changes therein, during the activation of immune signaling.

As a first step in our analysis, we evaluated the relationship among the metrics (i.e., angular differences, skewness of intensity distribution, and skeletonized pixel occupancy) measured from this image dataset to validate the efficacy of these metrics as benchmarks for our cluster analyses. As show in S1 Fig, the data demonstrated a weak correlation in each of the three pairwise combinations evaluated, with Pearson correlation coefficients of *r* = 0.12 in angular degrees versus skewness (S1A Fig), *r* = -0.3 in angular degrees versus occupancy (S1B Fig), and *r* = -0.15 in skewness versus occupancy (S1C Fig). These results show that the data of each metric changed independently, confirming that these are indeed acceptable metrics for hierarchical clustering of MF organization.

Once we established the robustness of our approach, above, we first evaluated the relationship(s) between stomatal aperture and the three metric parameters of actin MF configurations. As shown in S2 Fig, we observed a positive correlation between stomatal aperture and MF orientation, with a Pearson correlation coefficient of *r* = 0.29 in stomatal aperture versus angular degrees (S2A Fig), a very weak correlation with MF bundling (Pearson correlation coefficient: *r* = 0.06 in stomatal aperture versus skewness; S2B Fig), and a negative correlation with MF density (Pearson correlation coefficient: *r* = -0.16 in stomatal aperture versus occupancy; S2C Fig), although we did not observe a strong correlation in each of three pairwise combinations evaluated. In total, these data are in agreement with the previous work of Higaki et al. [15], which reported a similar trend during the diurnal cycling of Arabidopsis. These data support that this is a robust method for the evaluation of immune-induced temporal and spatial/organizational changes in stomata-localized actin MFs.

### Clustering of stomatal guard cells based on MF configurations

**Fig 2:**
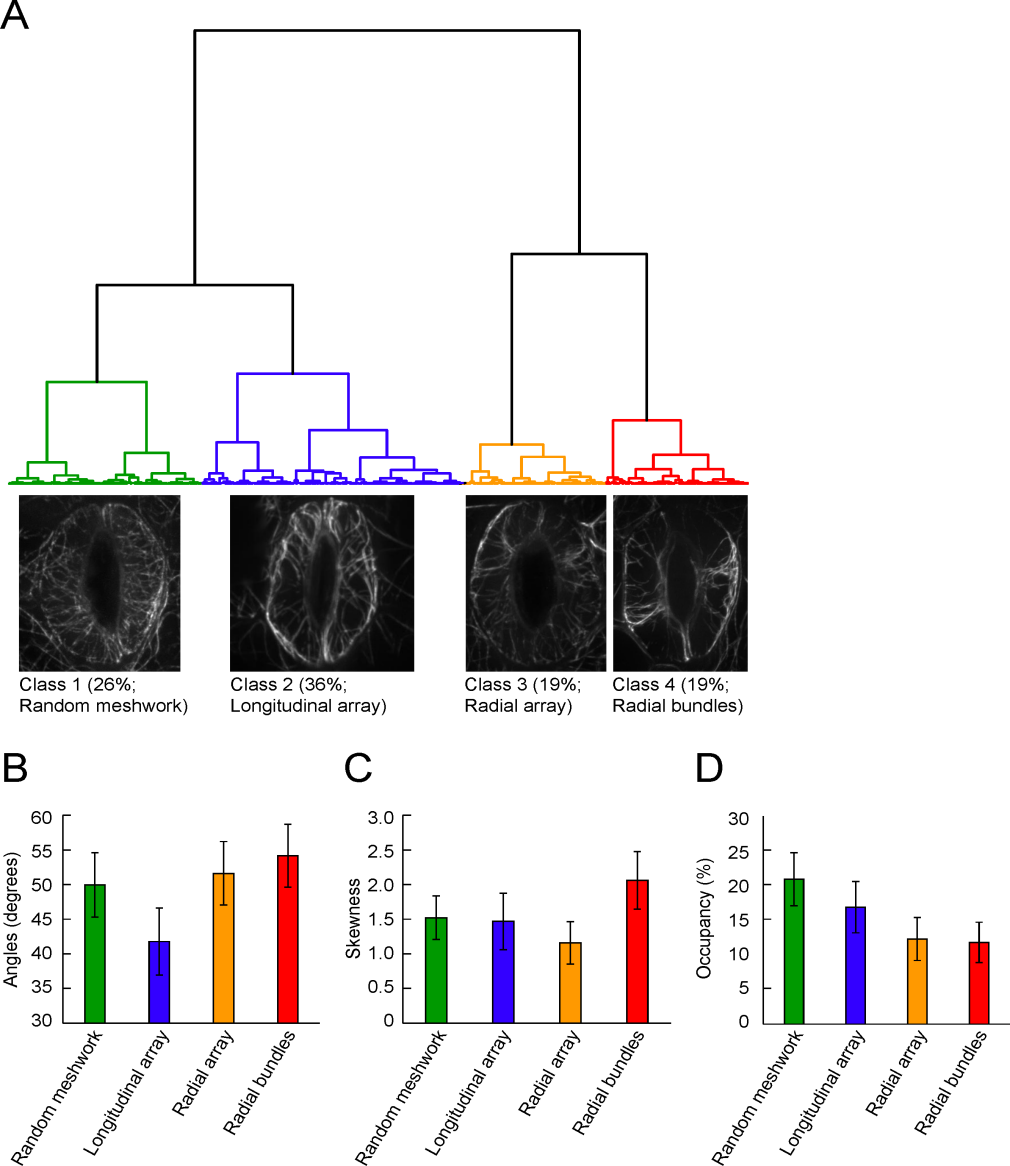
Quantitative evaluation, hierarchical cluster analysis and classification of cytoskeletal configurations in guard cells. (A) Cluster dendrogram of 1793 guard cell pairs for MF configuration assignment. Data were analyzed for a total of 1793 pairs of guard cells that were image-acquired across treatment and time, including *Pseudomonas syringae* pv. *tomato* DC3000 inoculation, flg22 elicitor treatment, chitin elicitor treatment, and mock inoculations for pathogen and elicitor treatments. Also included was a temporal analysis (every 2 hours) during a 24 h diurnal cycle. Collected images were classified into four classes based on the patterns of the metrics of MF orientation, MF bundling and MF density: class 1 (random mesh works), class 2 (longitudinal array), class 3 (radial array) and class 4 (radial bundles). Representative images of class 1, 2, 3, and 4 filament orientations are shown. Characterization of each class obtained by cluster analysis of 1793 MF configurations in (B) mean angular differences between MFs and the nearest stomatal pore edge (metrics for MF orientation), (C) skewness of GFP-fABD2 fluorescence intensity distribution (metrics for MF bundling), and (D) occupancy of skeletonized MF pixels in guard cell regions (i.e., metrics for MF density).

Image clustering analysis based on the three metric parameters revealed that all 1,793 images were classified into four classes: class 1 (26%, highlighted in green), class 2 (36%, highlighted in blue), class 3 (19%, highlighted in orange), and class 4 (19%, highlighted in red) (Fig 2A). Percentages indicate the proportion of each class within the total population. For cataloging and descriptive classification of actin MF configurations during diurnal and immune stimulus induction, we termed class 1 (shown in green) as ‘random meshwork’, based on a quantitative evaluation that revealed the majority of these filament arrays consisted of a random orientation with a high density metric. Class 2 (shown in blue) was defined as ‘longitudinal arrays’, based on the lowest mean of angular degrees, while class 3 (shown in orange) was defined as a ‘radial array’ because the quantitative majority of the MFs were arranged in a radial orientation. Lastly, class 4 (shown in red) filaments were cataloged as ‘radial bundles’ based on the observation that the majority of actin MFs arranged in a radial orientation had a high degree of intensity distribution skewness. Mean values of the classes in each metric parameter of actin MF configurations, including mean angular differences between MFs and the nearest stomatal pore edge (metrics for MF orientation, Fig 2B), skewness of GFP-fABD2 fluorescence intensity distribution (metrics for MF bundling, Fig 2C), and occupancy of skeletonized MF pixels in guard cell regions (metrics for MF density, Fig 2D) were also assessed.

### Pathogen inoculation specifically perturbs stomatal actin microfilament configuration

**Fig 3:**
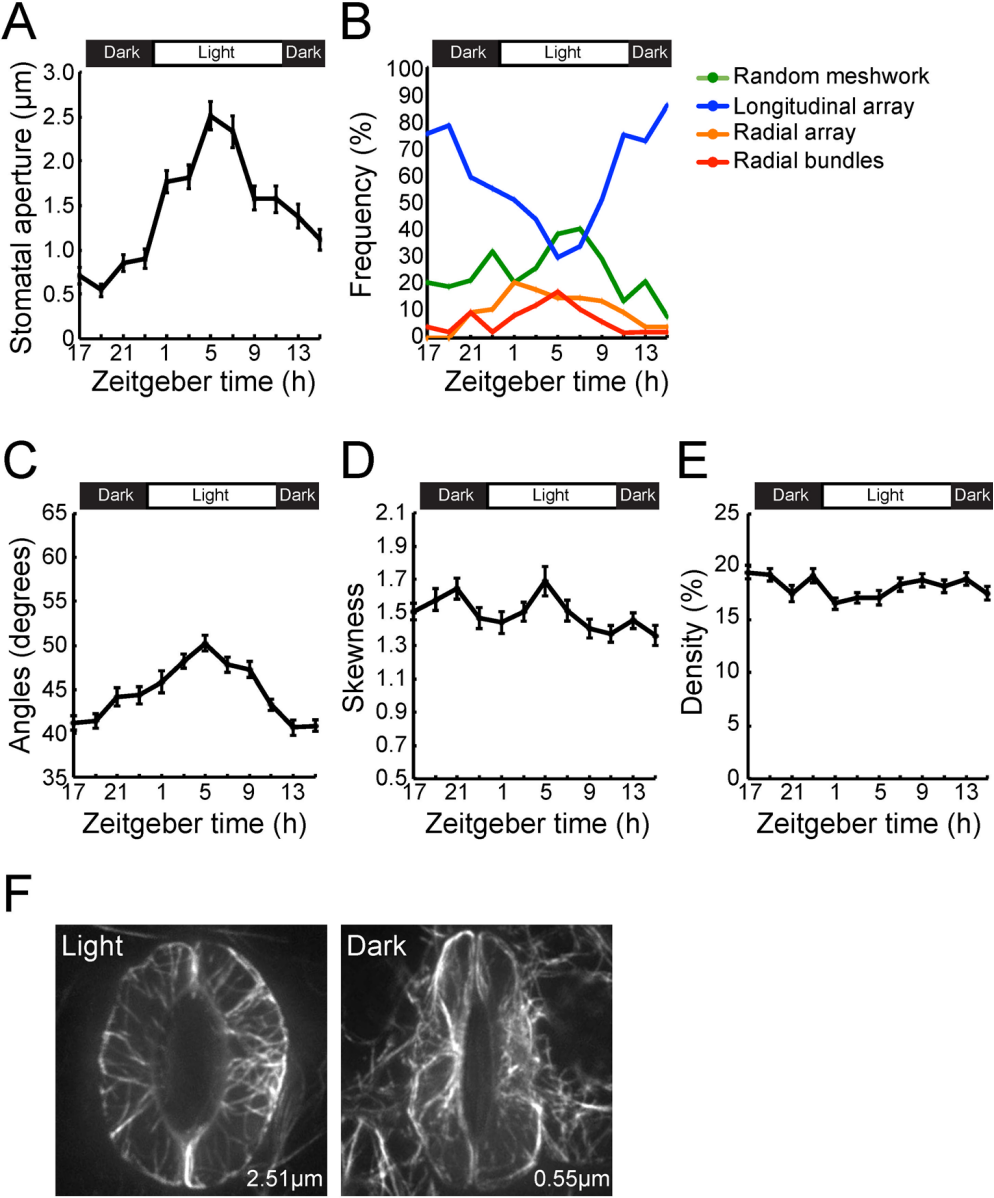
Evaluation of microfilament configurations and stomatal apertures over a diurnal cycle. (A) Changes in stomatal aperture at 2-hour increments over a 24 h (12 h light/12 h dark) diurnal cycle. (B) Changes in population distributions of each class of actin MF configuration obtained by hierarchical cluster analysis over a 12 h light/12 h dark cycle. Changes in actin MF configuration over a 12 h light/12 h dark diurnal cycle in metrics for (C) orientation, (D) bundling, and (E) density of MFs. (F) Representative images of stomatal guard cells collected by spinning disc confocal microscopy during light and dark periods over a 24 h diurnal cycle. Images were collected at 5 h Zeitgeber time (lights on, stomata maximally opened) and 19 h Zeitgeber time (lights off, stomata maximally closed), respectively. The averaged values of apertures at each time point are shown inside images. Error bars represent means ± SEM from stomatal aperture and metrics for MF orientation, bundling and density. Data was collected from 3 independent biological repeats (n = 42–52).

Perception of the virulent bacterial pathogen *Pst* DC3000 by Arabidopsis induces rapid changes in stomata aperture, including stomata closure within 1 hpi. Within 4 hpi, re-opening of the host stomata is induced through the activity of the bacterial phytopathogen toxin coronatine [22]. To define the relationship between the guard cell actin MF response during infection and the activation of host immunity, we first asked if pathogen infection changes the naïve diurnal patterning of MF configurations in stomatal guard cells. To do this, we first quantified the proportion of each of the 4 classes of actin MF configurations over a 24 h diurnal cycle (12 h light/12 h dark; sampling every 2 h; e.g., Fig 1B). As shown in Fig 3A, we observed a predictable pattern of guard cell gating over a 24 h diurnal cycle, with changes in the proposition of the 4 class of MF configurations over the 12 h light/ 12 h dark cycle (Fig 3B). Additionally, we identified temporal increases in angular degrees during the transition from dark to light (i.e., ca. 23–24 h Zeitgeber; Fig 3C), with a concomitant decrease in the intensity distribution skewness (Fig 3D). MF densities were relatively stable during diurnal rhythms (Fig 3E), and in parallel, we observed that the averaged values of MF orientation indicators in each image acquisition time also trended towards an enrichment in longitudinal orientation during dark periods (i.e., when stomata were closed; Fig 3A and 3C). In total, we did not identify a relationship between stomatal aperture and an indicator for MF bundling (Fig 3A and 3D), or MF density (Fig 3A and 3E) during the naïve diurnal cycling of stomatal guard cell gating. Proportional changes in the percentages of the 4 classes of MF configuration showed that radial array and bundle configurations emerged in open stomata (Fig 3B and 3F, ‘Light’) while longitudinal arrays were enriched in closed stomata (Fig 3B and 3F, ‘Dark’). In total, these data are in agreement with previous studies [15].

**Fig 4:**
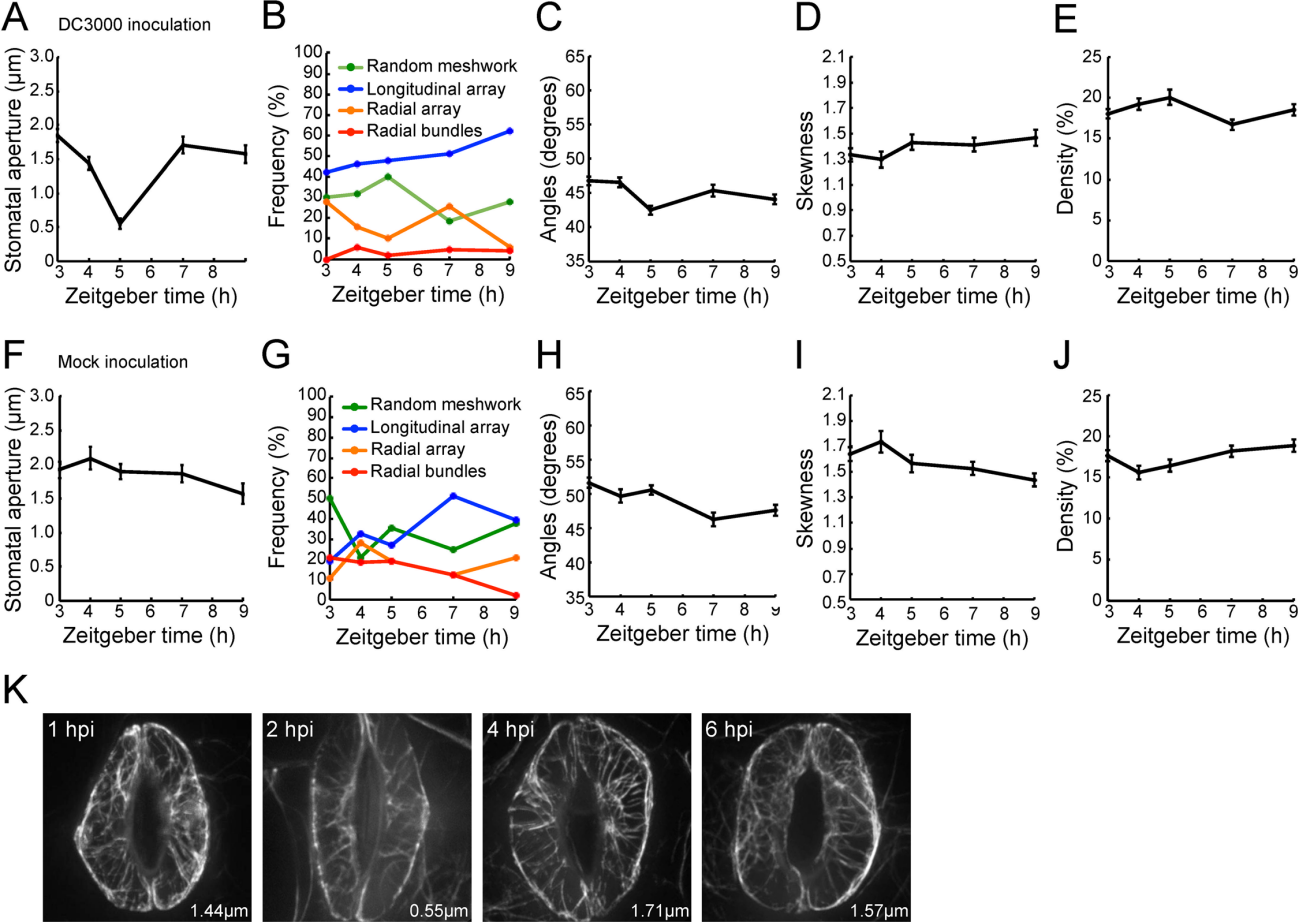
Evaluation of microfilament configurations and stomatal apertures following plant inoculation with a virulent bacterial pathogen. (A) Changes in stomatal aperture following *Pseudomonas syringae* pv. *tomato* DC3000 (*Pst* DC3000) inoculation. (B) Changes in population distributions of each class of actin MF configuration following *Pst* DC3000 inoculation as obtained by cluster analysis. Changes elicited in the guard cell actin MF array by *Pst* DC3000 in metrics for (C) orientation, (D) bundling, and (E) density of MFs. (F) Changes in stomatal aperture following mock inoculation, and (G) the concomitant changes in population distributions of each class of MF configuration obtained by cluster following mock inoculations. Changes induced by mock inoculation in (H) orientation, (I) bundling, and (J) density. Error bars represent mean ± SEM, for stomatal apertures and indicators for MF orientation, MF bundling, and MF density. Data was collected from 3 independent biological repeats (n = 43–50). (K) Representative images of stomatal guard cells from Col-0 plants expressing GFP-fABD2 following *Pst* DC3000 inoculation. Micrographs shown are representative images collected at 1 h, 2 h, 4 h, and 6 h post-inoculation. The averaged values of apertures at each time point are shown.

Once we established the diurnal behavior of guard cell-localized actin MF organization, we next examined changes in stomatal apertures and the configurations of MFs after *Pst* DC3000 inoculation (Fig 4A–4E), comparing the mock treatment (Fig 4F–4J). Eleven- to 13-day-old GFP-fABD2-expressing Arabidopsis seedlings were dip-inoculated with the virulent bacterial pathogen *Pst* DC3000, and guard cells images were collected by SDCM. As expected, we observed *Pst* DC3000-induced stomatal closing and re-opening (Fig 4A) as previously reported [22], while in mock (i.e., dH_2_O) inoculations, stomatal apertures were roughly constant over the time course of analysis (Fig 4F). As a function of guard cell MF organization with regard to the timing of bacteria inoculation-induced stomatal closing (i.e. until two hours after the inoculation) (3–5 h in Zeitgeber time), we observed decrease in the angular differences (i.e., an approximate 10 degrees reduction; Fig 4C, 3–5 h in Zeitgeber time), which parallels bacterial-induced stomatal closure (Fig 4A, 3–5 h in Zeitgeber time). Similarly, we did not identify a relationship between stomatal aperture and MF bundling (Fig 4A and 4D) and density (Fig 4A and 4E) from serial sections collected during bacterial infection. The relationship between stomatal apertures and MF configurations were similar in that in during diurnal cycling (Fig 2). The representative GFP-fABD2 images were shown in Fig 4K. In mock treatment, we did not observe significant changes in stomatal aperture and metrics for MF configurations (Fig 4F–4J).

Actin MF configuration changes were also assessed by appearance frequency in each class identified by clustering analysis (Fig 4B and 4G). In the timing of stomatal closure, longitudinal array and random meshwork configurations were dominant while radial array configurations decreased (from 28% to 10%) (Fig 4B, 3–5 h Zeitgeber time). In mock treatment samples, random meshwork decreased (from 50% to 35%) (Fig 4G, 3–5 h Zeitgeber time); however, both radial and longitudinal array configurations slightly increased (from 10% to 19% (radial array); from 19% to 27% (longitudinal array)) (Fig 4G, 3–5 h Zeitgeber time). These observations were consistent with decreased values of angular differences in the inoculated guard cells compared with mock treatment (Fig 4C and 4H). As a function of guard cell MF organization with regard to the timing of bacteria-induced re-opening of stomatal guard cells (i.e. ca. 2–4 hours after the inoculation; Fig 4A, 5–7 h Zeitgeber), we observed decrease in random meshwork (from 40% to 19%) and increase in radial array (from 10% to 26%) (Fig 4B, 5–7 h Zeitgeber time). In the mock treatment, we observed increase in longitudinal array (from 27% to 51%) (Fig 4G, 5–7 h Zeitgeber time). These observations were consistent with slight decrease in MF density in inoculated guard cells compared with mock inoculation (Fig 4E and 4J). Radial bundles were relatively constant (at low levels) in bacteria-inoculated samples (from 2% to 4%) (Fig 4B, 5–7 h Zeitgeber time), while they slightly decreased in mock samples (from 19% to 12%; Fig 4G, 5–7 h Zeitgeber time). These data are consistent with a changing trend of values of skewness of GFP-fABD2 fluorescent intensity distributions (Fig 4D and 4I). In total, our data support the conclusion that following pathogen perception, as well as under normal oscillation of the diurnal cycle, MFs undergo a longitudinal array configuration in guard cells, and this orientation coincides with guard cell closure. Moreover, these data demonstrate that induced changes in actin MFs in guard cells in response to pathogen perception are similar to those during the transition from light to dark during diurnal cycling.

### Evaluation of microfilament configurations and stomatal apertures during PAMP-triggered immune signaling

**Fig 5:**
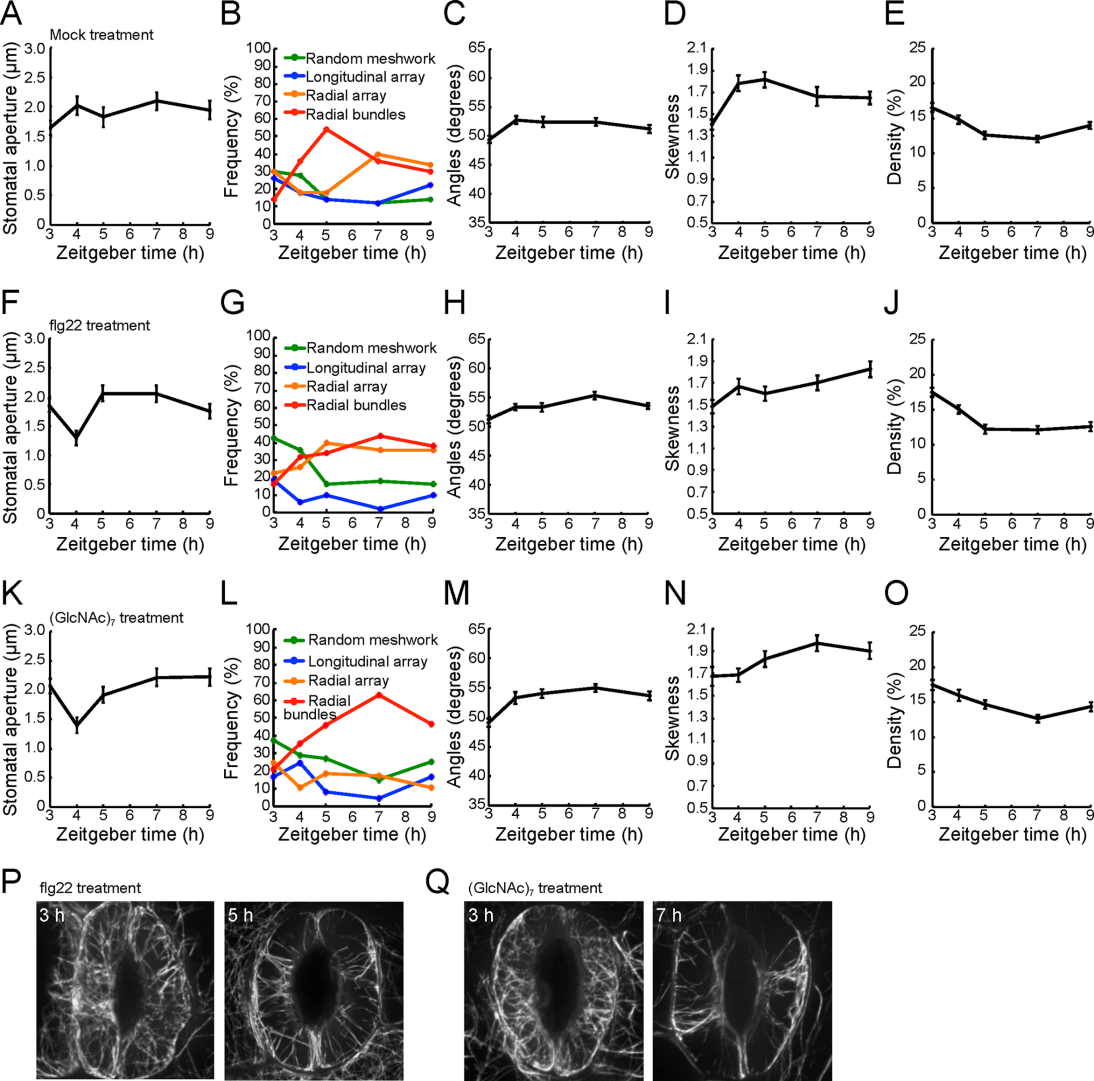
Evaluation of microfilament configurations and stomatal apertures following flg22 and chitin elicitation. (A) Changes in stomatal aperture following mock treatment. The proportion of MF configurations and population distributions of each class in stomatal guard cells following mock inoculation, as represented by (B) MF configuration population frequency and metrics for (C) orientation, (D) bundling, and (E) density of MFs. (F) Changes in stomatal aperture following flg22 elicitor treatment. Image collection began at 3 h Zeitgeber. Changes by flg22 elicitor treatment in population distributions of each class (G) obtained by cluster analysis based on metrics for (H) orientation, (I) bundling, and (J) density of MFs. (K) Changes in stomatal aperture following chitin oligosaccharide elicitor treatment. Changes in guard cell actin MF configurations elicited following chitin oligosaccharide elicitor treatment in (L) population distributions of each class obtained by cluster analysis of MF configurations, as evaluated by (M) orientation, (N) bundling, and (O) density of MFs. Error bars represent mean ± SEM from stomatal apertures, angle, skewness, and density of three independent biological repeats (n = 43–52).

Previous work has shown that pathogen elicitor treatment induces a rapid, yet transient, change in the actin MF density in epidermal pavement cells of Arabidopsis, presumably as a preamble to the activation of PTI [17]. To ask if this response is similar to bacterial pathogen-induced changes in the guard cell, and additionally, how the activation of immune signaling impacts MF orientation or bundling, we analyzed the guard cell actin MF response following pathogen elicitor perception. To do this, we collected images of guard cell pairs using SDCM following flg22 and chitin elicitation (Fig 1B). Using these images, we assessed the distributions of each metric parameter, including the frequency of each class, described above. In brief, 11- to 13-day-old GFP-fABD2-expressing Arabidopsis seedlings were elicited with 25 μM flg22 and serial images of guard cells were collected by SDCM. Mock-treated samples (i.e., dH_2_O only) were collected and analyzed following all elicitor treatments in parallel. After the microscopic image processing, we measured stomata apertures (Fig 5A and 5F), metrics for MF configurations (Fig 5C–5E and 5H–5J) and MF class frequencies (Fig 5B and 5G).

To correlate the guard cell actin MF response as a function of guard cell gating, we examined the relationship between aperture and the frequency of each MF cluster class following pathogen elicitor treatment. As shown in Fig 5F, flg22 treatment resulted in the rapid closure of stomata. Following elicitation of Arabidopsis seedlings with flg22, we identified radial array and radial bundles as the predominant populations in stomatal guard cells over the 6 h time course of treatment, with the approximate frequency of each class doubling over the time-course of treatment (from 22% to 36% (radial array); from 16% to 38% (radial bundles)) (Fig 5G, 3–9 h Zeitgeber time). As a function of the individual classes, we observed an increase in the proportion of MF configurations assuming a radial array conformation at 2 hpi following flg22 treatment (from 22% to 40%; Fig 5G, 3–5 h in Zeitgeber time), as compared to the same configuration following mock inoculation (from 30% to 18%) (Fig 5B, 3–5 h Zeitgeber time). Interestingly, this trend mirrored our observation of a concomitant change in radial bundles (from 14% to 54% (mock); from 16% to 34% (flg22 treatment)), which showed a 22% decrease at 2 hpi (compare Fig 5B–5G, 3–5 h in Zeitgeber time). As a function of flg22 induced changes in MF orientations, we did not observe a significant change over the time course of the experiment (compare Fig 5C–5H). However, as a function of the indicator for MF bundling, we observed decrease at 2 hpi (compare Fig 5D and 5I). Taken together, these data demonstrate that pathogen elicitor perception results in a rapid change in the higher ordered state of the actin MF array within the guard cell. Interestingly, and in contrast to previously published data describing the organization of actin filaments in epidermal cells following PAMP treatment [17], we did not observe significant changes in metrics for actin MF orientation and density in stomatal guard cells following flg22 treatment (orientation, Fig 5C versus Fig 5H; density, Fig 5E versus Fig 5J).

Next, to determine if elicitor-induced changes in the actin MF array is specific with regard to the PAMP itself, we treated 11- to 13-day-old Arabidopsis seedlings with purified chitin, a fungal-derived PAMP capable of eliciting the rapid induction of immune-associated processes in plants [23–25]. First, as an indicator for the biological activity of PAMP perception, chitin treatment induced the rapid closure of stomatal guard cells (Fig 5K, 3–4 h Zeitgeber time). Moreover, and similar to our observations with flg22-treated samples, we identified radial bundles as the predominant MF configuration at 4 and 6 hours following elicitor treatment (Fig 5L). Additionally, as was the case with flg22 treatment, above, we also did not observe any changes in metrics for MF orientation and bundling in stomatal guard cells following chitin treatment (orientation, Fig 5C versus Fig 5M; bundling, Fig 5D versus Fig 5N). However, and as a point of distinction between the frequencies of MF class accumulation following flg22 treatment and the diurnal cycling, we did not observe an increase in the frequency of radial array configurations over the time-course analyzed. Finally, we did not observe a change in MF density, as compared to mock treatment, following elicitation with chitin (Fig 5E versus Fig 5O). Representative guard cell images are shown for flg22 (Fig 5P) and chitin (Fig 5Q) elicitation.

## Discussion

In the current study, we employed high spatial and temporal resolution imaging methods for the visualization and quantification of pathogen-induced changes in actin filament organization in the stomatal guard cell. Coupled with an unguided hierarchical clustering analysis, we present the first comprehensive evaluation of induced changes in stomatal actin MF organization during pathogen infection and immune signaling. In short, we have identified organizational signatures in the MF array that are specifically associated with pathogen infection. We posit that these MF signatures represent important transitions in the stomatal guard cell response following stimulus perception and the activation of signaling.

Recent studies have demonstrated a role for the actin cytoskeleton in the activation of plant immunity following pathogen infection, including that as a required component of the plant immune system [4,17,18,20,26,27]. However, much of our understanding for a role of the plant actin cytoskeleton during pathogen infection is corollary, relying in large part on the use of pharmacological agents (i.e., cytochalasin D, latrunculin B) to associate inhibition of cytoskeletal dynamics with changes in cellular response and a subsequent block in immune signaling. Recent work aimed at clarifying a role for the cytoskeleton as an early signaling component of the plant immune system has revealed that changes in organization are rapid, yet transient. However, it is unclear whether these changes are part of the immune response, or are simply associated with specific, or non-specific, pathogen-induced responses during infection [21,28]. Indeed, in mammalian cells, stress perception has been demonstrated to induce the rapid and transient formation of actin rods, which can temporarily halt actin cytoskeletal remodeling, thus affecting numerous downstream signaling processes required for response to perturbations [3]. Similarly, in plants, a variety of immune-eliciting bacterial strains and purified pathogen elicitors have been demonstrated to induce rapid and transient increases in actin polymerization, a phenomenon that was observed only when the plant has its full complement of corresponding pattern recognition receptors [17,18].

Numerous studies have demonstrated that both actin and stomatal guard cells specifically respond to a diverse array of internal and external cues, including those associated with plant growth and development, and abiotic and biotic stress perception and signaling (reviewed in [29,30]). Herein, we extended initial work by Higaki et al. [15] to evaluate pathogen-induced changes in actin cytoskeletal organization by analyzing approximately 1800 guard cell pairs during both diurnal cycling and the activation of immune signaling to ask: Do pathogens perturb homeostatic oscillations in the host actin filament array during infection? Interestingly, our analysis identified a series of unique, stepwise higher order transitions in the actin MF array in plant stomatal guard cells in response to pathogen infection. In short, we observed that changes in the MF array in guard cells as a function of immune signaling are distinct from the diurnal patterns observed during light/dark transitions. In short, this approach enabled us to identify and characterize 4 MF classifications with respect to the perception of pathogen and specific pathogen-elicitor events. Notably, we observed that the immune-induced temporal accumulation and distribution of each of the 4 classes of actin MFs are distinct from the naïve diurnal closure response. Coupled with a recent study by Ou et al. [31], it is interesting to speculate that our observations of perturbations to the dynamics of the actin cytoskeleton are an important component of the guard cell perception and signaling mechanism(s) that underpins signal specificity and the activity of cellular processes in response to stress.

Quantitative evaluation of actin filament orientation, bundling, and density within stomata revealed that actin MFs are arranged into four major classes during diurnal and immune elicitation: class 1 (random meshwork), class 2 (longitudinal array), class 3 (radial array), and class 4 (radial bundles). As an extension of previous work by Higaki et al. [15], the data presented herein reveal that the proportion of each of these classes vary depending on the nature of the applied stimulus. For example, and as a point of similarity between diurnal and immunity-induced MF signatures, we observed the same patterning under untreated, naïve, conditions (open = radial; closed = longitudinal), as previously published [15]. However, during pathogen infection, we identified a pattern of filament reorganization that correlated with infection, as evidence by a specific disruption of the MF ‘radial array’. What remains unclears is if changes in the proportion and organization of the actin MF array is a consequence of pathogen virulence, or is a required modification to the naïve, incessant, cycling that is required for the activation of immunity? We posit that the current study establishes a foundation for addressing these questions, and through further work in this area, will serve to define the contribution and specificity of actin MF dynamics to stomatal-based processes, including for example, the relationship(s) between hormone and immune signaling as a function of actin.

The plant stomata represent a paradigm for the analysis of the pathogen-actin interface. For example, numerous studies have established that stomatal closure is an important output of innate immunity in plants [32,33]. In particular, the observation that stomata function in innate immunity and actively close in response to PAMPs and live bacteria provide a foundation to elucidate the core signaling components involved in stomatal immunity–defined as PAMP recognition and signaling associated with stomata closure and blocking of pathogen invasion and growth [22,34–36]. However, significant knowledge gaps still remain. Of relevance to the current study, and future work in this area, is a need to define the functional and mechanistic relationship(s) between the activation of immunity and the organization of the actin cytoskeleton. As a tractable system for addressing this, particularly with respect to the utilization of genetic mutants perturbed in stomatal function, we posit that stomatal guard cells represent an attractive cell type for further dissecting the role of actin dynamics during immune signaling.

## Materials and Methods

### Plant materials and growth conditions

*Arabidopsis thaliana* plants expressing GFP-fABD2 (Col-0/GFP-fABD2); [17]) were grown in an environmentally-controlled growth chamber at 22.5°C under a 12 h light/12 h dark (100 μE fluorescent light) cycle at 60% relative humidity.

### Pathogen strains and plant inoculation

*Pseudomonas syringae* pv. *tomato* DC3000 (*Pst* DC3000) was grown as previously described [4]. Arabidopsis seedlings (11–13 days post germination) were dip-inoculated with bacterial suspensions of 3 × 10^7^ colony-forming units (cfu) mL^-1^ as described by Kunkel et al. [37].

### Elicitor treatments

Treatment of Arabidopsis seedlings with flg22 (25 μM) or chitin ([GlcNAc]_7_); 40 μM) was performed as described by Lozano-Durán et al. [38], with slight modification. Eleven- to 13-day-old seedlings were used for flg22- and [GlcNAc]_7_-triggered stomatal closure experiments. Seedlings (11–13 days post-germination) were kept under constant light for 3 h to ensure uniformity across all plants for open stomata. Samples were elicited with flg22 or [GlcNAc]_7_ at 3 h Zeitgeber time by floating cotyledons in an elicitor solution such that the abaxial surface of the cotyledon was facing down into the solution. Comparisons to mock treatments (i.e., dH_2_O only) were included in all experiments.

### Spinning disc confocal microscopy

To observe actin filament organization in guard cells, Arabidopsis guard cells were imaged using a spinning disc confocal microscopy, collecting 0.5 μm interval serial sections, using an UPlanSApo 100x/1.40 objective lens on an Olympus IX70 and a CSU10 scanning head (Yokogawa Electric Corporation, Tokyo, Japan). To capture images of guard cell MFs, cotyledons were floated, with its abaxial surface up, on a glass slide covered with basal buffer [50 mM KCl, 5mM 2-(*N*-morpholine)-ethanesulphonic acid (MES)-Tris, pH 6.5, and 10 mM CaCl_2_], and then covered by a coverslip. For sample selection and analysis, a recent study by our group [39] demonstrates that both mature (ca. 4-6-week-old) plants and 11-14-day-old cotyledons show similar behavior and biological activity with elicited with biotic stressors (e.g., pathogens and purified pathogen elicitors)

### Image cluster analysis

Serial optical sectional images of GFP-fABD2-expressing Arabidopsis seedlings were used to quantitatively evaluate actin microfilament orientation, bundling, and density, as previously described [15]. To evaluate actin filament orientation, collected serial images were projected, binalized with Otsu’s thresholding, and skeletonized. Using the processed images, the mean angular difference between skeletonized actin microfilament pixel pairs and the nearest pixel pairs of the stomatal pore edges were segmented manually. To evaluate actin microfilament bundling, serial images were skeletonized and projected, and the skewness of the distribution of GFP-fABD2 fluorescence intensity in the processed images was used as an indicator of actin microfilament bundling. To evaluate actin microfilament density, the processed skeletonized GFP-fABD2 signal pixel numbers per manually segmented guard cell region pixels was calculated using the similarly processed image for skewness measurements. All image-processing procedures were performed using KBI plugins (http://hasezawa.ib.k.u-tokyo.ac.jp/zp/Kbi/HigStomata). For cluster analysis, data were classified using hierarchical clustering employing the statistical software R (http://www.r-project.org).

### Stomatal aperture measurements

Stomatal aperture measurements were performed using an estimate of the pixel number of stomatal pore width using projected images from each of the serial sections collected by spinning disc confocal microscopy. Stomata aperture measurements, derived from the exact pixel numbers (width), were used to calculate distances, as represented in micrometers (μm).

## Supporting Information

S1 Fig Comparison of quantitative values between each metrics. Correlation analyses between metrics for (A) microfilament (MF) orientation and MF bundling, (B) MF orientation and MF density, and (C) MF bundling and MF density. Data were plotted from 1793 pairs of guard cell images that were acquired by spinning disc confocal microscopy across all treatments and all time points. *r* indicates the Pearson correlation coefficient.
(TIFF)

S2 Fig Relationship between stomatal aperture and the three metric parameters. Correlation analyses between metrics for (A) MF orientation, (B) MF bundling, and (C) MF density. Data were plotted from 1793 pairs of guard cells images that were acquired across all treatments and all time points. *r* indicates the Pearson correlation coefficient.
(TIFF)
