# Supplementary figures and images for: Quantitative Evaluation of Stomatal Cytoskeletal Patterns during the Activation of Immune Signaling in Arabidopsis thaliana
Source: PLoS One. 2016 Jul 14;11(7):e0159291. doi: 10.1371/journal.pone.0159291 (PMC4944930; doi:10.1371/journal.pone.0159291)

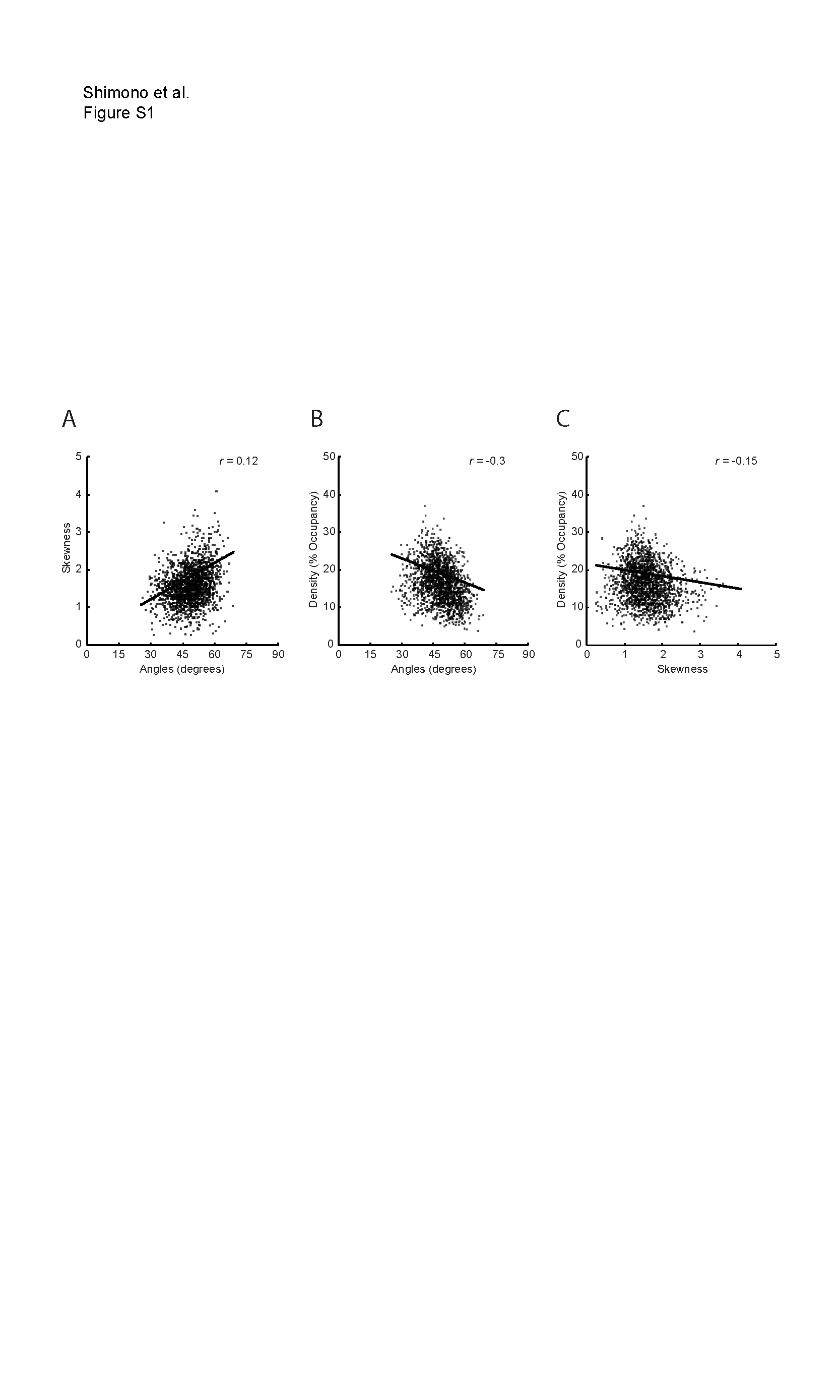

Supplement: S1 Fig — Correlation analyses between metrics for (A) microfilament (MF) orientation and MF bundling, (B) MF orientation and MF density, and (C) MF bundling and MF density. Data were plotted from 1793 pairs of guard cell images that were acquired by spinning disc confocal microscopy across all treatments and all time points. r indicates the Pearson correlation coefficient. (TIFF) [file pone.0159291.s001.tiff]

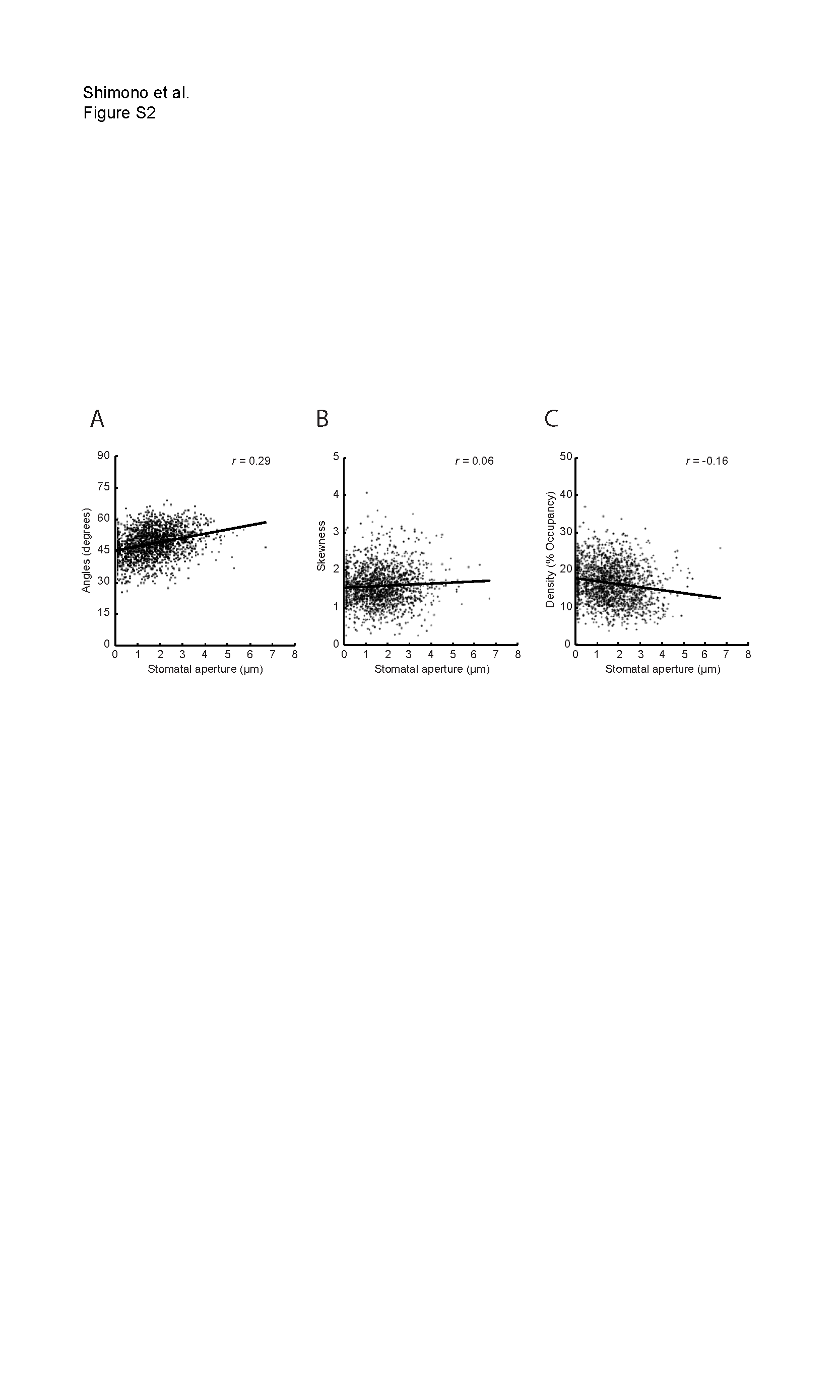

Supplement: S2 Fig — Correlation analyses between metrics for (A) MF orientation, (B) MF bundling, and (C) MF density. Data were plotted from 1793 pairs of guard cells images that were acquired across all treatments and all time points. r indicates the Pearson correlation coefficient. (TIFF) [file pone.0159291.s002.tiff]
